# The relationship between genetic variants associated with primary ovarian insufficiency and lipid profile in women recruited from MASHAD cohort study

**DOI:** 10.1186/s12905-021-01550-8

**Published:** 2022-01-07

**Authors:** Mohammad Reza Mirinezhad, Hamideh Ghazizadeh, Maliheh Aghsizadeh, Mohammad Zamiri Bidary, Alireza Naghipour, Elahe Hasanzadeh, Mahdiyeh Yaghooti-Khorasani, Ali Ebrahimi Dabagh, Mohammad Reza Shadmand Foumani Moghadam, Nazanin Sheikh Andalibi, Zeynab Naseri Far, Habibollah Esmaily, Gordon A. Ferns, Tayebeh Hamzehloei, Alireza Pasdar, Majid Ghayour-Mobarhan

**Affiliations:** 1 Department of Medical Genetics and Molecular Medicine, Faculty of Medicine, Mashhad University of Medical Sciences, Mashhad, Iran; 2 International UNESCO Center for Health-Related Basic Sciences and Human Nutrition, Mashhad University of Medical Sciences, Metabolic Syndrome Research Center, 99199-91766 Mashhad, Iran; 3 Student Research Committee, Mashhad University of Medical Sciences, Mashhad, Iran; 4 Department of Nutrition Sciences, Varastegan Institute for Medical Sciences, Mashhad, Iran; 5 Social Determinants of Health Research Center, Mashhad University of Medical Sciences, Mashhad, Iran; 6 Division of Medical Education, Brighton and Sussex Medical School, Falmer, Brighton, BN1 9PH Sussex UK; 7 Division of Applied Medicine, Medical School, University of Aberdeen, Foresterhill, Aberdeen, AB25 2ZD UK; 8 Metabolic Syndrome Research Center, Faculty of Medicine, Mashhad University of Medical Sciences, Mashhad, Iran

**Keywords:** Primary Ovarian Insufficiency, Cardiovascular disease, ARMS-PCR, ASO-PCR

## Abstract

**Background and aim:**

Primary Ovarian Insufficiency (POI) is defined by the occurrence of menopause before the age of 40 years. It is often associated with cardiovascular disease (CVD). The purpose of this study was to explore the relationship between POI-associated genotypes cardiometabolic disorder risk factors.

**Methods:**

One hundred seventeen women with POI and one hundred eighty-three healthy women without POI were recruited in this study. DNA was extracted and analyzed using ASO-PCR or Tetra ARMS-PCR. Lipid profiles were also assessed.

**Results:**

Multivariate logistic regression analysis showed that individuals with GG vs. TT genotype of the rs1046089 SNP were more likely to have a higher serum LDL (*p* = 0.03) compared to the control group. There was also a significant association between low serum HDL and rs2303369 and rs4806660 SNP genotypes in the POI group. In the POI group, the percentage of those with high total cholesterol was lower in those with a CC genotype compared to those with a TT genotype (*p* = 0.03).

**Conclusion:**

Some SNPs reported to be associated with POI appear to be independently associated with dyslipidemia. These results may be helpful to identify subjects with POI who may be susceptible to CVD.

## Introduction

Menopause is defined as occurring when menstruation stops for 12 consecutive months due to loss of ovarian follicular activity; this usually occurs around the age of 45–55 years [1]. Menopause that happens before the age of 40 years is called Primary Ovarian Insufficiency (POI) which may be natural or due to or reproductive surgeries [2–4]. This condition is accompanied by amenorrhea, estrogen deficiency and an increase of gonadotrophin levels [5]. It has been reported that about 3.6% of women develop POI [6]. Various factors such as smoking [7], certain medications [8], infections and genetic and autoimmune disorders have been associated with POI [9]. Iatrogenic causes such as radiotherapy, chemotherapy, pelvic surgery are also associated with POI [5, 9–14]. About 60–90% of POI cases are idiopathic [14]. Several studies have also found that POI increases the risk of hypertension [15], cardiovascular disease [3], osteoporosis [16] cerebral infarction [17], all-cause mortality [18] type 2 diabetes mellitus [15], and other negative health consequences [16].

Genetic factors also play a significant role in POI [19]. The heritability of menopausal age is estimated about 30–85% [20]. About 20–25% of POI cases are due to genetic causes [14]. Genome-wide association studies (GWAS) have identified a polymorphism (rs16991615) of minichromosome maintenance 8 homologous recombination repair factor (MCM8) gene involved in the age of natural menopause [21]. Also, rs1046089 and rs4806660, located on Proline Rich Coiled-Coil 2A (PRRC2A) and transmembrane (TMEM) gene, respectively are associated with the age at menopause [22, 23]. GWAS identified several other variants that are associated with POI [24].

Deleterious changes in risk factors for cardiometabolic disorders often occur around the age of menopausal [18, 25–27]. Estrogen is involved in dilating blood vessels and helping blood flow [18]. Various studies have also shown that estrogen therapy in postmenopausal women reduces serum total cholesterol and low density lipoprotein (LDL) cholesterol concentrations, and increases serum high-density lipoprotein (HDL) cholesterol and triglyceride concentrations [28, 29]. Moreover, lack of ovarian function in the menopause is involved in the activation of the renin-angiotensin system, leads to immunodeficiency, inflammation and endothelial dysfunction [18, 30]. These are associated with obesity, diabetes and high blood pressure [18, 30]. Several studies have shown that age at menopause is associated with cardiovascular disease [18, 31–33]. A Japanese study found women with early menopause had a higher risk for hypercholesterolemia [34], and another study showed that early menopause is associated with hypertension [35]. Sarnowski et al. founded that genetic variants associated with early menopause are also associated with increased cardiovascular disease risk [36]. There appeared to be a need to evaluate the relationship between POI-related variants with lipid profile and susceptibility to cardiometabolic disease risk factors. No previous study has investigated association between polymorphisms related to early menopause with lipid profiles.

Therefore, we aimed to explore the associations between POI-related variants with lipid profile and susceptibility to cardiometabolic disease risk factors in Mashhad stroke and heart atherosclerotic disorders (MASHAD) cohort study population.

## Methods

Totally, 117 women who had POI were included in the case group. Healthy women (n = 183) were recruited into the control group. All of participants were recruited as part of the MASHAD study. The MASHAD study was a cohort study from 2010–2020 that were included 9704 participants aged 35–65 years who will be follow-up exams every three years until 2020 [37]. The inclusion criteria were as follows: the diagnostic criterion was based on the definition of POI: (1) Women who go through menopause before the age of 40 years; (2) 12 continuous months have passed since the last bleeding; and (3) serum FSH > 40 IU/L. Exclusion criteria were: women over 40 years old, with a history of diseases and surgeries affecting menstruation (oophorectomy, hysterectomy), history of genetic abnormalities and syndromes associated with an early menopause is a part of their manifestation, history of using drugs affecting menstruation. Blood samples were collected into vacutainer® plain tubes, and were taken after 12 h fast. Blood samples were centrifuged at 4° C in 5000 rpm for 15 min and the serum part was used for lipid profile measurement. Body Mass Index (BMI) was measured using standard method [37]. Kidney, liver, and thyroid activity were normal in all participants.

### DNA extraction and quality controls

Participants' DNA was extracted from 200 µl blood or buffy coat samples using a DNA extraction kit (Pars Tous, Mashhad, Iran). Qualitative and quantitative quality control was performed by agarose gel electrophoresis (Pars Tous, Mashhad, Iran) and Nano drop 2000 (Thermo Fisher Scientific, USA) in 280 and 260 nm wavelengths, respectively.

### Allele-specific oligonucleotide polymerase chain reaction (ASO-PCR)

The ASO-PCR reaction volume was 15 µl which included: 1.5 µl water, 2 µl genomic DNA, 1 µl of each primer, and 7.5 µl master mix (Pars Tous, Mashhad, Iran). First, to carry out PCR, we performed one cycle of denaturation for 7 min at 95 °C. After that, 35 cycles include the following: 95 °C for 30 s, annealing for 30 s at 60 °C, 72 °C for 30 s, and eventually one cycle of 7 min was done for final extension.

### Tetra amplification refractory mutation system PCR (ARMS-PCR)

Tetra ARMS was carried out by the same method and the same composition of 15 µl reaction volume that performed in ASO-PCR. Primers were designed with Primer1 software [38].

### Lipid profile measurements and dyslipidemia diagnosis

Total cholesterol (TC), triglycerides (TG), and high-density lipoprotein cholesterol (HDL-C) and low-density lipoprotein cholesterol (LDL-C) levels were measured from serum which was taken from participants 12 h fasting using standard method [39, 40]. The NCEP ATPIII criteria [41] were used to diagnose dyslipidemia. (1) If serum cholesterol levels ≥ 200 mg /dl (5.2 mmol/l) is considered hypercholesterolemia. (2) If HDL cholesterol levels < 50 mg/dl (< 1.3 mmol/l) for women is considered low HDL cholesterol. (3) If LDL cholesterol levels ≥ 130 mg/dl (≥ 3.4 mmol/l) is considered high LDL cholesterol. (4) If serum triglycerides ≥ 150 mg/dl is considered Hypertriglyceridemia.

### Physical activity level assessment

The equations of James and Schofield for energy requirements, were used to assess physical activity of all participants. Questions regarding the physical activity level were based on the mentioned equations which were selected from World Health Organization MONICA project questionnaires [42]. The level of physical activity was calculated by total energy expenditure (TTE) and basal metabolism rate (BMR) during a whole day and night [43].

### Ethics

All steps of the study were approved by the Mashhad University of Medical Sciences (MUMS) Ethics Committee. Informed consents were obtained from all subjects and the procedure and possible risks were explained completely, pursuant to the Declaration of Helsinki [44].

### Statistical analysis

All analysis tests were performed by Statistical Package for Social Sciences (SPSS) (IBM Corp. Released 2016. IBM SPSS Statistics for Windows, Version 24.0. Armonk, NY: IBM Corp.). The values in this study have been reported frequently with percentage or mean and standard deviation. Single Nucleotide Polymorphisms' (SNPs') genotypes between POI cases and healthy controls were compared by Chi-square test. Assessment of normal distribution in quantitative data was performed by Kolmogorov-Smirnoff test. Man-Whitney test was used for comparing quantity of normally distributed values between the subgroups. In this study with having at least 117 subjects in each group and confidence interval 95% and power 80% the value of the standardized effect size about 37% reported which has a good precision. Besides, we used multivariate logistic regression to prevent confounder's factors from affecting our results. P-value < 0.05 was statistically significant.

## Results

The clinical characteristics of the population have been summarized in Table 1. In our cross-sectional analysis, participants had a mean age of 55 years averagely. The mean age of the case and control group were 55.21 ± 5.56 and 54.62 ± 2.89, respectively. There was not significantly difference (P = 0.4). The control-women were matched by age. The details on the genotype distribution of these SNPs between the two groups was investigated in our previous studies [45]. All of the polymorphisms were in Hardy–Weinberg equilibrium.

**Table 1:** Demographic features and characteristics of the study population

Characteristics	POI cases, N = 117	Controls, N = 183	*P*-value
Age (Y)	55.21 ± 5.56	54.62 ± 2.89	0.4
BMI (kg/m^2^)	28.78 ± 5.06	29.34 ± 4.22	0.323
FBG (mg/dl)	88.74 ± 20.06	88.96 ± 31.43	0.948
PAL	1.78 ± 0.25	1.70 ± 0.23	**0.008**
Non smoker	86 (74.8%)	146 (79.8%)	0.390
Ex-smoker	10 (8.7%)	9 (4.9%)	
Current smoker	19 (16.5%)	28 (15.3%)	
TC (mg/dl)	207.8 ± 35.8	188.8 ± 33.1	**< 0.001**
TG (mg/dl)	111.0 (82.0–162.5)	117.0 (80.0–159.0)	0.684
LDL-C (mg/dl)	128.39 ± 33.96	110.47 ± 33.42	**< 0.001**
HDL-C (mg/dl)	47.45 ± 9.33	45.06 ± 11.34	**0.040**

Statistically significant values are shown in bold

Data are shown as Mean ± SD; Student t-test and Chi-square test were used; POI: Primary Ovarian Insufficiency; SD: Standard Deviation; BMI: Body Mass Index; FBG: Fasting Blood Glucose; PAL: Physical Activity Level; TC: Total Cholesterol; TG: Triglyceride; HDL: High Density Lipoprotein; LDL: Low Density Lipoprotein

Differences in level of lipid serum between different genotypes of POI-related polymorphisms were examined (Table 2). In the POI cases, serum total cholesterol was significantly different between various rs16991615, rs244715, rs4806660, and rs10183486 SNP genotypes; however, this difference was not observed in the healthy controls. Interestingly, three of four of the investigated factors including serum total cholesterol, triglyceride, and HDL were substantially difference in different genotypes of rs4806660 SNP in participants with POI and this association was not detected in controls group.

**Table 2:** Association of genotypes related to POI with lipid profile in target population divided into POI cases and healthy controls

Polymorphisms	POI cases (N = 117)					Controls (N = 183)			
rs16991615	AA	GA	GG	*P*	AA		GA	GG	*P*
Serum total cholesterol (mg/dl)	222.3 ± 41.4^a^	195.8 ± 35.3^b^	209.4 ± 29.5^ab^	**0.01**	190.2 ± 33.8		191.2 ± 33.5	186.4 ± 32.7	0.64
Triglyceride (mg/dl)	108.0 (78.0–181.0)	112.0 (82.0–147.0)	107.0 (89.0–156.0)	0.96	98.5 (65.8–121.3)		129.0 (87.0–159.0)	108.0 (79.5–162.3)	0.14
HDL (mg/dl)	48.6 ± 7.7	45.9 ± 8.9	48.0 ± 10.5	0.44	45.0 (40.8–53.0)		44.0 (35.0–53.0)	43.0 (35.6–54.3)	0.58
LDL (mg/dl)	136.1 ± 37.8	120.3 ± 29.9	130.6 ± 34.1	0.15	116.7 ± 35.2		112.7 ± 34.0	107.1 ± 32.5	0.39

rs244715	GG	AG	AA	*P*	GG	AG	AA	*P*
Serum total cholesterol (mg/dl)	213.3 ± 44.4^ab^	216.7 ± 35.5^a^	198.8 ± 32.3^b^	**0.04**	185.8 ± 25.8	182.9 ± 29.2	192.1 ± 35.0	0.21
Triglyceride (mg/dl)	140.5 (77.5–202.7)	112.0 (85.0–179.0)	108.0 (82.7–151.5)	0.75	107.0 (84.0–231.3)	122.0 (85.5–163.5)	113.0 (77.0–149.5)	0.55
HDL (mg/dl)	46.3 ± 8.6	47.8 ± 8.2	47.4 ± 10.5	0.88	43.5 (33.8–49.3)	42.0 (36.3–55.7)	44.0 (35.6–52.9)	0.93
LDL (mg/dl)	126.8 (111.9–158.8)	134.4 (115.9–146.5)	124.1 (93.1–142.6)	0.18	96.1 ± 36.4^ab^	97.2 ± 34.3^a^	118.2 ± 30.5^b^	** < 0.001**

rs451417	AA	CA	CC	*P*	AA	CA	CC	*P*
Serum total cholesterol (mg/dl)	201.0 (191.0–236.0)	207.5 (178.0–231.7)	208.5 (181.2–223.2)	0.71	194.5 (174.0–211.5)	181.5 (164.5–201.5)	192.0 (162.0–217.0)	0.31
Triglyceride (mg/dl)	120.0 (82.0–185.0)	111.5 (78.3–153.0)	107.5 (84.5–151.5)	0.51	106.0 (88.3–144.0)	124.0 (77.3–166.5)	115.0 (80.0–154.0)	0.94
HDL (mg/dl)	47.5 ± 9.1	46.2 ± 9.0	48.4 ± 9.8	0.56	45.5 (39.3–54.4)	43.5 (34.3–54.5)	42.8 (36.6–52.1)	0.78
LDL (mg/dl)	132.3 ± 38.3	122.0 ± 29.8	130.7 ± 33.9	0.39	117.4 ± 28.9	104.7 ± 34.1	113.9 ± 33.3	0.14

rs1046089	AA	GA	GG	*P*	AA	GA	GG	*P*
Serum total cholesterol (mg/dl)	196.0 (177.0–225.0)	207.0 (184.0–232.0)	209.0 (186.5–224.5)	0.76	177.1 ± 23.4	192.5 ± 33.1	188.0 ± 35.0	0.13
Triglyceride (mg/dl)	112.0 (79.0–214.0)	112.0 (82.0–153.0)	109.0 (84.0–154.5)	0.87	112.0 (80.0–155.0)	118.0 (84.0–162.5)	117.0 (76.0–159.0)	0.55
HDL (mg/dl)	50.4 ± 7.7	47.4 ± 9.4	46.6 ± 9.7	0.49	52.0 (40.0–58.0)^a^	45.0 (34.0–55.0)^ab^	43.0 (35.1–50.0)^b^	**0.03**
LDL (mg/dl)	122.5 ± 40.1	127.2 ± 32.8	132.2 ± 34.7	0.65	75.5 (58.6–111.0)^a^	108.0 (87.1–137.0)^b^	122.5 (101.1–138.6)^bc^	** < 0.001**

rs7246479	AA	TA	TT	*P*	AA	TA	TT	*P*
Serum total cholesterol (mg/dl)	201.5 (196.3–233.5)	200.5 (176.0–225.5)	213.0 (200.0–234.0)	0.12	178.6 ± 21.3	187.2 ± 31.8	191.3 ± 35.1	0.48
Triglyceride (mg/dl)	101.5 (73.3–167.0)	108.0 (81.3–150.7)	118.0 (94.0–186.0)	0.29	145.0 (93.5–164.3)	118.5 (84.7–152.3)	107.0 (77.0–161.0)	0.63
HDL (mg/dl)	48.7 ± 7.1	47.6 ± 9.2	46.8 ± 10.4	0.83	53.5 (41.5–57.7)	44.0 (35.0–52.5)	42.8 (36.0–53.1)	0.26
LDL (mg/dl)	137.9 ± 31.2	123.8 ± 30.7	133.6 ± 39.8	0.23	82.6 ± 29.4^a^	107.8 ± 33.9^ab^	115.5 ± 32.0^b^	**0.02**

rs4806660	CC	TC	TT	*P*	CC	TC	TT	*P*
Serum total cholesterol (mg/dl)	200.6 ± 34.8^ab^	217.0 ± 34.3^a^	199.8 ± 35.9^b^	**0.04**	161.4 ± 25.1	189.1 ± 33.2	190.6 ± 32.9	0.08
Triglyceride (mg/dl)	93.5 (73.3–168.0)^ab^	122.0 (97.5–183.5)^a^	100.0 (73.0–140.0)^b^	**0.02**	133.0 (100.0–167.0)	111.5 (80.7–160.5)	117.0 (76.7–156.0)	0.66
HDL (mg/dl)	53.5 ± 12.3^a^	44.9 ± 8.0^b^	48.7 ± 9.0^ab^	**0.01**	46.0 (40.0–56.0)	43.7 (35.5–54.3)	43.2 (35.7–52.5)	0.82
LDL (mg/dl)	112.2 ± 32.9	134.7 ± 34.9	125.6 ± 32.1	0.08	73.1 ± 26.1^a^	104.3 ± 33.0^b^	118.6 ± 31.4^c^	** < 0.001**

rs10183486	TT	CT	CC	*P*	TT	CT	CC	*P*
Serum total cholesterol (mg/dl)	197.7 ± 27.7^ab^	199.3 ± 34.6^a^	220.1 ± 35.9^b^	**0.01**	193.9 ± 35.3	192.4 ± 36.5	185.2 ± 29.5	0.32
Triglyceride (mg/dl)	97.5 (74.7–119.5)	107.5 (80.7–150.7)	122.0 (94.0–185.0)	0.12	143.0 (91.0–180.5)	111.0 (77.5–144.0)	120.0 (82.5–164.5)	0.23
HDL (mg/dl)	47.2 ± 6.4	47.7 ± 11.2	47.2 ± 7.5	0.95	35.5 (31.4–39.3)^a^	44.0 (39.0–54.5)^b^	43.5 (34.0–53.3)^bc^	**0.02**
LDL (mg/dl)	118.9 ± 25.1^ab^	119.5 ± 31.1^a^	140.9 ± 35.5^b^	**0.003**	120.5 ± 29.3	114.4 ± 34.3	106.1 ± 32.7	0.17

rs2303369	TT	CT	CC	*P*	TT	CT	CC	*P*
Serum total cholesterol (mg/dl)	206.5 ± 29.7	213.6 ± 36.8	200.8 ± 36.5	0.23	181.9 ± 19.6	192.6 ± 32.8	185.9 ± 35.3	0.19
Triglyceride (mg/dl)	98.0 (69.0–129.0)	117.0 (94.5–182.0)	106.0 (77.5–152.0)	0.05	106.0 (75.0–150.0)	120.0 (84.5–162.3)	117.0 (76.0–154.5)	0.36
HDL (mg/dl)	52.0 ± 10.1^a^	44.6 ± 8.5^b^	48.9 ± 9.1^ab^	**0.01**	56.5 (49.8–60.8)^a^	44.5 (34.0–55.0)^b^	42.8 (35.3–48.7)^bc^	**0.002**
LDL (mg/dl)	117.6 (100.9–139.6)	134.4 (116.3–149.0)	123.6 (103.1–147.8)	0.14	85.8 ± 27.6^a^	109.7 ± 32.2^b^	117.1 ± 33.2^bc^	**0.001**

Statistically significant values are shown in bold

POI: Primary Ovarian Insufficiency; HDL: High-Density Lipoprotein; LDL: Low Density Lipoprotein; One-way analysis of variance (ANOVA) OR Kruskal–Wallis tests were used;  Significant *P* value < 0.05

Furthermore, Tables 3 and 4 show the results of multivariate logistic regression analysis before and after adjustment for age and physical activity level. These results for rs1046089 showed individuals with GG genotype were more likely to have low LDL risk (OR = 5.48, CI = 1.14–26.34, *p* = 0.02) than individuals with the AA genotype in control group using a multivariate logistic regression test. Also, the results demonstrate that there was a significant association with high TG risk in CC variant vs. TT in rs10183486 in POI group (OR = 4.63, CI = 1.17–18.29, *p* = 0.02). Furthermore, these results suggest that individuals carried the recessive homozygous genotype (CC) of rs2303369 SNP compared to individuals with dominant homozygous genotype (TT) had an increased risk of a high serum LDL, low serum HDL and the risk of high TC was decreased in control group.

**Table 3:** Investigation of the relationship between the risk of polymorphisms related to Primary Ovarian Insufficiency and dyslipidemia factors in the study population in a non-adjusted model

Polymorphisms Lipid profile abnormalities	POI cases				Controls			
	OR (95% CI)	*P*	OR (95% CI)	*P*	OR (95% CI)	*P*	OR (95% CI)	*P*
rs16991615	GA/AA		GG/AA		GA/AA		GG/AA	
High LDL (mg/dl)	0.65 (0.24, 1.74)	0.39	1.15 (0.45,2.97)	0.77	0.65 (0.23, 1.84)	0.42	0.46 (0.16,1.30)	0.14
High Triglyceride (mg/dl)	0.51 (0.17, 1.50)	0.22	0.87 (0.32, 2.35)	0.79	1.93 (0.51, 7.32)	0.33	2.41 (0.64, 9.02)	0.19
Low HDL (mg/dl)	1.43 (0.52, 3.89)	0.48	1.08 (0.41, 2.80)	0.87	0.96 (0.32, 2.85)	0.94	0.88 (0.30, 2.59)	0.82
High total cholesterol (mg/dl)	0.36 (0.12, 1.02)	0.054	0.89 (0.32, 2.51)	0.83	0.64 (0.23, 1.83)	0.41	0.63 (0.22, 1.78)	0.39

rs244715	AG/GG		AA/GG		AG/GG		AA/GG	
High LDL (mg/dl)	2.06 (0.57, 7.47)	0.27	1.03 (0.29, 3.69)	0.95	1.22 (0.13, 11.47)	0.85	3.16 (0.35, 28.01)	0.30
High Triglyceride (mg/dl)	0.42 (0.11, 1.54)	0.19	0.38 (0.10, 1.38)	0.14	1.12 (0.19, 6.66)	0.89	0.66 (0.11, 3.83)	0.64
Low HDL (mg/dl)	0.61 (0.16, 2.34)	0.48	0.78 (0.21, 2.93)	0.72	0.38 (0.04, 3.47)	0.39	0.35 (0.04, 3.11)	0.34
High total cholesterol (mg/dl)	1.86 (0.52, 6.94)	0.35	0.76 (0.21, 2.72)	0.68	0.71 (0.12, 4.26)	0.71	1.31 (0.23, 7.47)	0.75

rs451417	CA/AA		CC/AA		CA/AA		CC/AA	
High LDL (mg/dl)	1.23 (0.47, 3.26)	0.66	1.80 (0.71, 4.52)	0.21	0.53 (0.18, 1.55)	0.25	1.19 (0.43, 3.28)	0.73
High Triglyceride (mg/dl)	0.69 (0.25, 1.91)	0.48	0.55 (0.21, 1.48)	0.24	1.96 (0.59, 6.48)	0.27	1.52 (0.46, 5.02)	0.48
Low HDL (mg/dl)	1.27 (0.47, 3.42)	0.62	0.94 (0.37, 2.35)	0.89	0.82 (0.29, 2.30)	0.71	1.19 (0.42, 3.33)	0.73
High total cholesterol (mg/dl)	0.88 (0.33, 2.35)	0.80	1.07 (0.42, 2.75)	0.87	0.57 (0.20, 1.59)	0.28	1.01 (0.37, 2.72)	0.98

rs1046089	GA/AA		GG/AA		GA/AA		GG/AA	
High LDL (mg/dl)	2.58 (0.62, 10.62)	0.18	3.50 (0.79, 15.34)	0.09	4.82 (1.05, 21.98)	0.04	6.83 (1.48, 31.46)	0.01
High Triglyceride (mg/dl)	0.46 (0.12, 1.69)	0.24	0.57 (0.14, 2.27)	0.43	1.04 (0.38, 2.83)	0.92	0.77 (0.27, 2.18)	0.63
Low HDL (mg/dl)	1.68 (0.46, 6.10)	0.42	2.21 (0.56, 8.67)	0.25	1.76 (0.70, 4.44)	0.22	3.21 (1.20, 8.53)	0.02
High total cholesterol (mg/dl)	1.92 (0.53, 6.95)	0.32	2.21 (0.56, 8.67)	0.25	2.33 (0.79, 6.85)	0.12	1.83 (0.60, 5.55)	0.28

rs7246479	TA/AA		TT/AA		TA/AA		TT/AA	
High LDL (mg/dl)	0.46 (0.13, 1.61)	0.22	1.36 (0.35, 5.24)	0.64	2.86 (0.33, 24.54)	0.33	3.93 (0.46, 33.38)	0.21
High Triglyceride (mg/dl)	1.04 (0.25, 4.29)	0.95	2.25 (0.51, 9.76)	0.27	0.34 (0.07, 1.49)	0.15	0.43 (0.10, 1.87)	0.26
Low HDL (mg/dl)								
High total cholesterol (mg/dl)	0.53 (0.14, 1.93)	0.34	1.68 (0.40, 7.09)	0.47	1.44 (0.27, 7.63)	0.66	1.85 (0.35, 9.71)	0.46

rs4806660	TC/CC		TT/CC		TC/CC		TT/CC	
High LDL (mg/dl)	2.72 (0.72, 10.21)	0.13	1.63 (0.43, 6.13)	0.47	–	–	–	–
High Triglyceride (mg/dl)	1.87 (0.45, 7.76)	0.38	0.97 (0.22, 4.18)	0.97	0.61 (0.12, 2.96)	0.54	0.45 (0.09, 2.19)	0.32
Low HDL (mg/dl)	7.40 (1.75, 31.16)	0.01	3.68 (0.88, 15.27)	0.07	073 (0.13, 4.01)	0.72	0.73 (0.13, 4.02)	0.72
High Total Cholesterol (mg/dl)	2.14 (0.57, 7.92)	0.25	0.63 (0.17, 2.26)	0.48	–	–	–	–

rs10183486	CT/TT		CC/TT		CT/TT		CC/TT	
High LDL (mg/dl)	0.82 (0.23, 2.94)	0.76	2.71 (0.74, 9.92)	0.13	1.95 (0.38, 10.02)	0.42	1.43 (0.27, 7.33)	0.66
High Triglyceride (mg/dl)	1.75 (0.34, 8.98)	0.50	3.39 (0.66, 17.25)	0.14	0.33 (0.08, 1.37)	0.12	0.65 (0.16, 2.61)	0.55
Low HDL (mg/dl)	0.62 (0.16, 2.32)	0.48	0.80 (0.21, 3.06)	0.75	0.23 (0.02, 1.98)	0.18	0.20 (0.02, 1.72)	0.14
High Total Cholesterol (mg/dl)	1.50 (0.42, 5.34)	0.52	4.58 (1.21, 17.35)	0.02	1.30 (0.30, 5.60)	0.72	0.90 (0.21, 3.87)	0.89

rs2303369	CT/TT		CC/TT		CT/TT		CC/TT	
High LDL (mg/dl)	2.41 (0.82, 7.11)	0.11	1.34 (0.43, 4.10)	0.61	6.77 (0.85, 53.83)	0.07	9.06 (1.13, 72.25)	0.03
High Triglyceride (mg/dl)	3.23 (0.83, 12.49)	0.08	2.20 (0.54, 8.99)	0.26	1.42 (0.42, 4.80)	0.56	1.05 (0.30, 3.64)	0.93
Low HDL (mg/dl)	6.03 (1.92, 18.94)	0.002	2.51 (0.79, 7.89)	0.11	4.94 (1.47, 16.55)	0.01	9.83 (2.82, 34.27)	< 0.001
High Total Cholesterol (mg/dl)	0.75 (0.23, 2.44)	0.64	0.30 (0.09, 1.01)	0.05	2.88 (0.76, 10.86)	0.11	2.08 (0.54, 7.98)	0.28

Binary logistic regression models were performed; POI: Primary Ovarian Insufficiency; LDL: Low-Density Lipoprotein; HDL: High-Density Lipoprotein; High LDL > 130 and Normal LDL < 130; High Triglyceride > 150 and Normal Triglyceride < 150; Low HDL < 40 (Male) or < 50 (Female) and Normal HDL > 40 (Male) or > 50 (Female), High Total Cholesterol > 200 and Normal Total Cholesterol < 200; Normal groups of all lipid profile measures were considered as references; The common genotypes of each studied variants were considered as references; Significant *P*-value < 0.05

**Table 4:** Investigation of the relationship between the risk of polymorphisms related to Primary Ovarian Insufficiency and dyslipidemia factors in the study population in an adjusted model for age and physical activity level

Polymorphisms Lipid profile abnormalities	POI cases				Control			
	OR (95% CI)	*P*	OR (95% CI)	*P*	OR (95% CI)	*P*	OR (95% CI)	*P*
rs16991615	GA/AA		GG/AA		GA/AA		GG/AA	
High LDL (mg/dl)	0.63 (0.23, 1.72)	0.36	1.21 (0.46, 3.17)	0.69	0.67 (0.22, 2.04)	0.48	0.37 (0.12, 1.15)	0.08
High Triglyceride (mg/dl)	0.45 (0.15, 1.38)	0.16	0.84 (0.30, 2.33)	0.74	1.38 (0.34, 5.53)	0.64	1.69 (0.43, 6.61)	0.45
Low HDL (mg/dl)	1.37 (0.50, 3.76)	0.54	1.05 (0.40, 2.76)	0.91	0.63 (0.19, 2.05)	0.45	0.59 (0.18, 1.89)	0.38
High Total Cholesterol (mg/dl)	0.34 (0.12, 0.99)	0.04	0.91 (0.32, 2.57)	0.86	0.67 (0.22, 2.02)	0.48	0.55 (0.18, 1.65)	0.29

rs244715	AG/GG		AA/GG		AG/GG		AA/GG		
High LDL (mg/dl)	2.13 (0.58, 7.82)	0.25	1.02 (0.28, 3.69)	0.96	1.18 (0.12, 11.32)	0.88		3.11 (0.34, 27.94)	0.31
High Triglyceride (mg/dl)	0.39 (0.10, 1.48)	0.17	0.36 (0.09, 1.36)	0.13	1.44 (0.23, 8.86)	0.69		0.74 (0.12, 4.44)	0.75
Low HDL (mg/dl)	0.60 (0.15, 2.30)	0.46	0.78 (0.21, 2.96)	0.72	0.48 (0.05, 4.45)	0.52		0.39 (0.04, 3.53)	0.41
High Total Cholesterol (mg/dl)	1.88 (0.50, 7.04)	0.35	0.76 (0.21, 2.72)	0.68	0.68 (0.11, 4.21)	0.68		1.28 (0.22, 7.38)	0.78

rs451417	CA/AA		CC/AA		CA/AA		CC/AA		
High LDL (mg/dl)	1.27 (0.47, 3.42)	0.63	1.80 (0.71, 4.58)	0.21	0.52 (0.17, 1.55)	0.24		0.99 (0.35, 2.83)	0.99
High Triglyceride (mg/dl)	0.57 (0.19, 1.63)	0.29	0.51 (0.18, 1.39)	0.19	1.84 (0.54, 6.30)	0.32		1.34 (0.39, 4.60)	0.64
Low HDL (mg/dl)	1.18 (0.43, 3.22)	0.74	0.91 (0.36, 2.32)	0.85	0.72 (0.24, 2.10)	0.55		1.09 (0.37, 3.18)	0.87
High Total Cholesterol (mg/dl)	0.86 (0.31, 2.35)	0.77	1.06 (0.41, 2.73)	0.89	0.56 (0.19, 1.60)	0.28		0.84 (0.30, 2.35)	0.75

rs1046089	GA/AA		GG/AA		GA/AA		GG/AA		
High LDL (mg/dl)	2.52 (0.61, 10.45)	0.20	3.62 (0.82, 16.09)	0.09	3.64 (0.75,17.59)	0.10		**5.89 (1.22, 28.34)**	**0.02**
High Triglyceride (mg/dl)	0.45 (0.11, 1.71)	0.24	0.59 (0.14, 2.42)	0.47	0.64 (0.21, 1.92)	0.42		0.46 (0.15, 1.45)	0.18
Low HDL (mg/dl)	1.72 (0.47, 6.25)	0.41	2.31 (0.58, 9.15)	0.23	1.33 (0.48, 3.67)	0.58		2.42 (0.85, 6.84)	0.09
High Total Cholesterol (mg/dl)	1.90 (0.52, 6.92)	0.33	2.26 (0.57, 8.93)	0.24	1.75 (0.55, 5.53)	0.33		1.53 (0.48, 4.88)	0.47

rs7246479	**TA/AA**		**TT/AA**		**TA/AA**		**TT/AA**		
High LDL (mg/dl)	0.45 (0.12, 1.62)	0.22	1.47 (0.37, 5.78)	0.58	1.97 (0.22, 17.75)	0.54		**2.42 (0.26, 22.31)**	0.43
High Triglyceride (mg/dl)	0.96 (0.22, 4.12)	0.96	2.27 (0.50, 10.30)	0.28	0.15 (0.02, 0.88)	0.03		0.15 (0.02, 0.95)	0.04
Low HDL (mg/dl)	1.37 (0.39, 4.77)	0.61	1.65 (0.43, 6.30)	0.45	4.27 (0.75, 24.30)	0.10		5.79 (0.98, 33.99)	0.05
High Total Cholesterol (mg/dl)	0.51 (0.14, 1.91)	0.32	1.74 (0.40, 7.41)	0.45	0.99 (0.17, 5.59)	0.99		1.14 (0.19, 6.62)	0.88

rs4806660	**TC/CC**		**TT/CC**		**TC/CC**		**TT/CC**		
High LDL (mg/dl)	2.64 (0.68, 10.16)	0.15	1.56 0.40, 6.14)	0.52	–	–		–	–
High Triglyceride (mg/dl)	1.55 (0.35, 6.76)	0.55	0.70 (0.15, 3.24)	0.65	0.44 (0.08, 2.44)	0.35		0.32 (0.06, 1.77)	0.19
Low HDL (mg/dl)	**6.91 (1.61, 29.50)**	**0.01**	3.32 (0.78, 14.17)	0.10	0.62 (0.10, 3.66)	0.60		0.60 (0.10, 3.50)	0.57
High Total Cholesterol (mg/dl)	1.95 (0.51, 7.45)	0.32	0.55 (0.14, 2.11)	0.39	–	–		–	–

rs10183486	**CT/TT**		**CC/TT**		**CT/TT**		**CC/TT**		
High LDL (mg/dl)	0.68 (0.18, 2.54)	0.57	2.36 (0.63, 8.86)	0.20	2.01 (0.38, 10.70)	0.41		1.29 (0.24, 6.85)	0.76
High Triglyceride (mg/dl)	1.68 (0.31, 8.92)	0.54	3.51 (0.67, 18.40)	0.13	0.35 (0.08, 1.49)	0.15		0.66 (0.16, 2.73)	0.57
Low HDL (mg/dl)	0.62 (0.16, 2.38)	0.49	0.82 (0.21, 3.20)	0.77	0.26 (0.3, 2.22)	0.22		0.21 (0.02, 1.84)	0.16
High Total Cholesterol (mg/dl)	1.43 (0.39, 5.21)	0.58	**4.43 (1.14, 17.16)**	**0.03**	1.32 (0.29, 5.83)	0.71		0.81 (0.18, 3.57)	0.78

rs2303369	**CT/TT**		**CC/TT**		**CT/TT**		**CC/TT**		
High LDL (mg/dl)	2.21 (0.74, 6.60)	0.15	1.24 (0.39, 3.87)	0.71	4.77 (0.57, 39.79)	0.15		7.48 (0.90, 61.75)	0.06
High Triglyceride (mg/dl)	3.22 (0.81, 12.85)	0.09	1.95 (0.46, 8.17)	0.36	0.89 (0.24, 3.35)	0.87		0.65 (0.17, 2.49)	0.53
Low HDL (mg/dl)	**6.19 (1.93, 19.88)**	**0.002**	2.39 (0.74, 7.66)	0.14	**4.03 (1.12, 14.48)**	**0.03**		**7.97 (2.19, 28.96)**	**0.002**
High Total Cholesterol (mg/dl)	0.68 (0.20, 2.27)	0.54	**0.27 (0.07, 0.93)**	**0.03**	2.12 (0.52, 8.55)	0.28		1.72 (0.42, 6.93)	0.44

Statistically significant values are shown in bold

Binary logistic regression models were performed; POI: Primary Ovarian Insufficiency; LDL: Low-Density Lipoprotein; HDL: High-Density Lipoprotein; High LDL > 130 and Normal LDL < 130; High Triglyceride > 150 and Normal Triglyceride < 150; Low HDL < 40 (Male) or < 50 (Female) and Normal HDL > 40 (Male) or > 50 (Female), High Total Cholesterol > 200 and Normal Total Cholesterol < 200; Normal groups of all lipid profile measures were considered as references; The common genotypes of each studied variants were considered as references; Adjusted with Age & Physical Activity Level; Significant *P*-value < 0.05

The risk of low HDL was increased in individuals carrying rs23303369 variant (CT) compared to non-carriers (TT) in both studied groups, (OR, 6.6; 95% CI, 1.88–19.53, *P* = 0.003 in POI group) and (OR = 4.21, CI = 1.17–15.18, *p* = 0.02 in control group). Table 5 shows the association of genotypes related to POI with lipid profile in the POI cases and healthy controls.

**Table 5:** Association of different genotypes related to POI with lipid profile in target population divided into POI cases and healthy controls

Polymorphisms									
rs16991615	AA			GA			GG		
	POI cases (N = 48)	Controls (N = 86)	*P*	POI cases (N = 40)	Controls (N = 79)	*P*	POI cases (N = 29)	Controls (N = 18)	*P*
Serum total cholesterol (mg/dl)	207.00 (50.00)	190.00 (57.00)	**0.020**	195.79 ± 35.28	191.16 ± 33.51	0.489	209.40 ± 29.54	186.42 ± 32.73	** < 0.001**
Triglyceride (mg/dl)	108.00 (103.00)	98.50 (56.00)	0.187	112.00 (65.00)	129.00 (72.00)	0.286	107.00 (67.00)	108.00 (83.00)	0.785
HDL (mg/dl)	48.62 ± 7.73	46.92 ± 8.39	0.489	45.94 ± 8.94	44.59 ± 12.12	0.538	48.00 (13.80)	43.00 (18.68)	0.120
LDL (mg/dl)	136.10 ± 37.79	116.74 ± 35.22	0.091	120.32 ± 29.94	112.70 ± 34.01	0.236	130.64 ± 34.13	107.10 ± 32.51	** < 0.001**

rs244715	GG			AG			AA		
	POI cases (N = 56)	Controls (N = 116)	*P*	POI cases (N = 49)	Controls (N = 61)	*P*	POI cases (N = 12)	Controls (N = 6)	*P*
Serum total cholesterol (mg/dl)	213.33 ± 44.41	185.83 ± 25.77	0.183	**211.00 (35.00)**	182.00 (40.00)	** < 0.001**	198.81 ± 32.30	192.13 ± 35.05	0.237
Triglyceride (mg/dl)	140.50 (125.00)	107.00 (147.00)	0.779	112.00 (94.00)	122.00 (78.00)	0.683	108.00 (69.00)	113.00 (73.00)	0.937
HDL (mg/dl)	46.30 ± 8.58	42.83 ± 8.56	0.430	48.00 (12.00)	42.00 (19.40)	0.114	47.37 ± 10.48	44.92 ± 11.33	0.181
LDL (mg/dl)	132.17 ± 39.17	96.13 ± 36.42	0.078	134.40 (30.59)	102.09 (50.51)	** < 0.001**	121.72 ± 32.17	118.16 ± 30.53	0.487

rs451417	AA			CA			CC		
	POI cases (N = 48)	Controls (N = 87)	*P*	POI cases (N = 36)	Controls (N = 76)	*P*	POI cases (N = 33)	Controls (N = 20)	*P*
Serum total cholesterol (mg/dl)	201.00 (45.00)	194.50 (38.00)	**0.038**	203.28 ± 33.02	183.95 ± 33.74	**0.024**	205.24 ± 33.68	192.12 ± 33.95	**0.005**
Triglyceride (mg/dl)	120.00 (103.00)	106.00 (56.00)	0.435	111.50 (75.00)	124.00 (89.00)	0.815	107.50 (67.00)	115.00 (74.00)	0.619
HDL (mg/dl)	47.46 ± 9.07	46.01 ± 8.85	0.576	46.19 ± 9.01	45.19 ± 12.64	0.632	48.65 (13.30)	42.80 (15.50)	**0.040**
LDL (mg/dl)	132.29 ± 38.32	117.44 ± 28.86		122.02 ± 29.80	104.74 ± 34.13		130.73 ± 33.94	113.86 ± 33.33	

rs1046089	AA			GA			GG		
	POI cases (N = 37)	Controls (N = 71)	*P*	POI cases (N = 69)	Controls (N = 89)	*P*	POI cases (N = 11)	Controls (N = 23)	*P*
Serum total cholesterol (mg/dl)	196.00 (48.00)	178.00 (40.00)	**0.038**	208.08 ± 36.16	192.53 ± 33.14	**0.006**	207.86 ± 35.61	188.02 ± 35.04	**0.006**
Triglyceride (mg/dl)	196.00 (48.00)	178.00 (40.00)	0.439	208.08 ± 36.16	192.53 ± 33.14	0.214	207.86 ± 35.61	188.02 ± 35.04	0.906
HDL (mg/dl)	50.41 ± 7.72	50.65 ± 11.57	0.952	47.80 (11.75)	45.00 (21.00)	0.195	46.90 (11.65)	43.00 (14.90)	0.090
LDL (mg/dl)	122.53 ± 40.11	85.01 ± 30.07	**0.005**	127.19 ± 32.75	109.64 ± 32.47	**0.001**	132.22 ± 34.72	119.75 ± 31.49	0.062

rs7246479	AA			TA			TT		
	POI cases (N = 36)	Controls (N = 89)	*P*	POI cases (N = 67)	Controls (N = 86)	*P*	POI cases (N = 14)	Controls (N = 8)	*P*
Serum total cholesterol (mg/dl)	213.75 ± 34.18	178.63 ± 21.30	**0.019**	201.86 ± 33.72	187.20 ± 31.81	**0.007**	216.94 ± 38.71	191.34 ± 35.06	**0.001**
Triglyceride (mg/dl)	101.50 (94.00)	145.00 (71.00)	0.427	108.00 (70.00)	118.50 (68.00)	0.420	118.00 (92.00)	107.00 (84.00)	0.144
HDL (mg/dl)	48.69 ± 7.12	50.75 ± 9.96	0.596	47.56 ± 9.17	45.18 ± 11.67	0.161	47.80 (13.10)	42.80 (17.15)	0.258
LDL (mg/dl)	137.98 ± 31.21	82.58 ± 29.38	**0.001**	123.89 ± 30.71	107.86 ± 33.90	**0.003**	134.80 (36.18)	120.00 (41.54)	**0.016**

rs4806660	CC			TC			TT		
	POI cases (N = 50)	Controls (N = 94)	*P*	POI cases (N = 53)	Controls (N = 82)	*P*	POI cases (N = 14)	Controls (N = 7)	*P*
Serum total cholesterol (mg/dl)	200.58 ± 34.85	161.43 ± 25.05	**0.019**	211.00 (35.00)	188.00 (36.00)	**< 0.001**	199.80 ± 35.90	190.63 ± 32.89	0.128
Triglyceride (mg/dl)	114.67 ± 59.99	132.71 ± 34.67	0.479	122.00 (86.00)	11.50 (80.00)	0.132	100.00 (67.00)	117.00 (79.00)	0.244
HDL (mg/dl)	53.50 ± 12.28	46.71 ± 9.32	0.225	46.85 (10.15)	43.75 (18.78)	0.860	48.70 ± 9.05	44.70 ± 11.72	**0.039**
LDL (mg/dl)	112.22 ± 32.96	73.08 ± 26.05	**0.016**	133.05 (34.01)	105.40 (52.35)	**< 0.001**	125.64 ± 32.13	118.66 ± 31.37	0.213

rs10183486	TT			CT			CC		
	POI cases (N = 47)	Controls (N = 93)	*P*	POI cases (N = 55)	Controls (N = 81)	*P*	POI cases (N = 15)	Controls (N = 9)	*P*
Serum total cholesterol (mg/dl)	197.67 ± 27.73	193.96 ± 35.32	0.790	199.31 ± 34.62	192.44 ± 36.45	0.276	220.13 ± 35.86	185.20 ± 29.52	** < 0.001**
Triglyceride (mg/dl)	102.92 ± 40.22	142.11 ± 54.65	0.073	107.50 (70.00)	111.00 (67.00)	0.973	122.00 (91.00)	120.00 (82.00)	0.494
HDL (mg/dl)	47.18 ± 6.38	36.35 ± 8..69	**0.004**	47.40 (13.72)	44.00 (15.50)	0.564	47.80 (11.40)	43.50 (19.30)	0.132
LDL (mg/dl)	118.99 ± 25.14	120.46 ± 29.33	0.903	119.54 ± 31.13	114.42 ± 34.31	0.380	138.20 (37.90)	106.10 (47.60)	** < 0.001**

rs2303369	TT			CT			CC		
	POI cases (N = 42)	Controls (N = 77)	*P*	POI cases (N = 54)	Controls (N = 90)	*P*	POI cases (N = 21)	Controls (N = 16)	*P*
Serum total cholesterol (mg/dl)	206.47 ± 29.72	181.88 ± 19.56	**0.008**	213.64 ± 36.80	192.59 ± 32.84	**0.001**	200.85 ± 36.50	185.90 ± 35.28	**0.032**
Triglyceride (mg/dl)	106.47 ± 52.80	108.38 ± 43.58	0.909	117.00 (88.00)	120.00 (78.00)	0.446	106.00 (75.00)	117.00 (79.00)	0.735
HDL (mg/dl)	52.01 ± 10.13	54.68 ± 11.40	0.466	45.00 (10.05)	44.50 (21.00)	0.937	48.80 (12.10)	42.80 (13.35)	**0.001**
LDL (mg/dl)	117.45 ± 29.31	82.78 ± 27.60	**0.001**	134.40 (32.72)	108.04 (48.80)	**0.001**	124.88 ± 33.16	117.08 ± 33.22	0.227

Statistically significant values are shown in bold

POI: Primary Ovarian Insufficiency; HDL: High-Density Lipoprotein; LDL: Low Density Lipoprotein; T-Student Test or Mann Whitney Test were used; Significant *P* value < 0.05

## Discussion

Our findings suggest that serum levels of several parameters in the fasted serum lipid profile consisting of total cholesterol, LDL, and HDL, but not serum triglycerides, were associated with POI. Further analyses indicated that genotypes of polymorphisms, which were previously reported to be related with the incidence of POI, are substantially associated with the level of lipid profile factors in POI cases. Moreover, we found that some genotypes in specific polymorphisms including rs4806660, rs10183486, and rs2303369 SNPs were significantly related to abnormalities regarding total cholesterol, LDL and HDL level in the cases' serum.

Initially our results found significant difference between POI cases and control participants for serum HDL, LDL, and total cholesterol levels, and these factors were significantly higher in the POI group. Gulhan and his coworkers have also found that among the 4 lipid factors, only total cholesterol and LDL were substantially different between cases diagnosed with premature ovarian failure (POF) and control subjects. POF group in this study had higher levels of total cholesterol and LDL [46]. Gulhan et al*.* work had included only women with previous history of successful childbirth and without any hormone therapy within the last 6 months, this inconsistency in HDL serum level had happened. However, this difference might have occurred due to their use of a small sample size as it was as one third as our study or due to the role of age as a confounding factor and also the role of some genetic variants related to POI. Interestingly, a recent study on 3 Dutch university medical centers did not report any significant difference in lipid profile between previously POI-diagnosed participants and population-based controls [47]. In their study, secondary amenorrhea (cessation of menstruation for at least 3 consecutive months) was one of the criteria for including POI cases; While, this period was too short compared to our criteria (12 consecutive months) and this important thing might have affected their results. Moreover, they have not indicated whether their participants had any previous history of surgeries, diseases, or taking medications related to female reproductive tract or not; Thus, this factor might have not been considered in their study which cause this disagreement.

Knauff et al*.* have reported changes in lipid profile of cases with POF compared to the controls is potentially related to the rate of ovarian function [48]. Another study which included cases who enter menopause by surgical ovariectomy, clarified that this intervention on female's reproductive tract, has caused impaired lipid metabolism 6 months post-surgery and substantial increase in all four lipid indicators (Total Cholesterol, Triglyceride, LDL, and HDL) [49]. Moreover it has been indicated that Free Androgen Index (FAI) was significantly lower in POI cases than people with regular menstrual cycles; while, it had been previously suggested that higher FAI was associated with high serum triglyceride and LDL in POI cases [50]. Finally, these results suggest that impairment in sexual hormones level, as a result of decreased females' reproductive system activity, is related to weaken lipid metabolism.

Our study has for the first time investigated the association between POI-related polymorphisms' genotypes and lipid profile status. In our analysis, we observed that genotypes of 2 different SNPs including rs4806660 (TMEM150B) and rs10183486 (TLK1) are substantially associated with abnormalities of lipid profile. Regarding the first one, low level of HDL was observed by 5.26 times higher in POI cases with TC genotype rather than CC participants. This association was not found in the controls. It is possible that these results are due to hormonal disorders that occur in postmenopausal individuals and consequently the lipid profile in individuals is impaired. Furthermore, for the second mentioned SNP, hypercholesterolemia was found over 450 percent higher in cases who carried CC than those with TT genotype. Previous studies have reported that both rs4806660 [51] and rs10183486 [22] polymorphisms are associated with the incidence of POI. While it has been proved that TLK1 gene encodes nuclear serine-threonine kinases [22] in which actively transfers signals from receptors of estrogen within the cell membrane to the nucleus [52], the exact function of TMEM150B gene product, called transmembrane protein 150B, has not yet been discussed.

Several SNPs, have been found to be associated with POI, a condition in which ovaries stop releasing sexual hormones, especially estrogen, making females infertile as early as before the age of 40. This estrogen hormone deficiency affects metabolism of lipids and thus, POI cases might face some lipid profile abnormalities that could increase the risk of cardiovascular disorders in participants of our study.

Our study was mainly limited by its design as a cross-sectional study, and changes in the level of lipid profile factors were not prospectively assessed. Data regarding pattern of the target population diet for the consumption of oils and meats, as the most common lipids, were not recorded in this study. We suggest future studies consider these limitations to achieve more reliable results. Moreover, several POI-related SNPs were not investigated in our study that might be associated with the cases' metabolic status and it is highly recommended to include these polymorphisms in the analysis.

In conclusion, SNPs which are previously found to be attributed to POI, could cause impairment in cases' lipid profile status through hormonal abnormalities. Present study clarifies that TC, CC and, CT genotypes of rs4806660, rs10183486, and rs2303369 SNPs, respectively increase the rate of dyslipidemia by approximately 5 times compared to their reference genotype (rs4806660: CC; rs10183486: TT; rs2303369: TT) in POI cases. But, GG and CC genotypes of rs1046089 and rs2303369 SNPs, respectively increase the rate of dyslipidemia about 6 times compared to their reference genotype in healthy population.

## Data Availability

The data that support the findings of this study are available from the corresponding author upon reasonable request.
